# Preparation of Cellulose-Activated Carbon Gel with High Activated Carbon Content and Its Adsorption of Methylene Blue

**DOI:** 10.3390/nano15110799

**Published:** 2025-05-26

**Authors:** Ung-Jin Kim

**Affiliations:** Department of Convergent Biotechnology and Advanced Materials Science, College of Life Sciences, Kyung Hee University, 1732 Deogyeong-daero, Giheung-gu, Yongin-si 17104, Gyeonggi-do, Republic of Korea; sbpujkim@khu.ac.kr

**Keywords:** cellulose, activated carbon, gel, LiBr aqueous solution, adsorbent

## Abstract

Activated carbon is a useful adsorbent for the removal of pollutants from the aqueous phase. In this study, an easy method to overcome the difficulty in separating activated carbon from a solution after adsorption has been developed. Cellulose-activated carbon gels with a high activated carbon content up to 70% in the total solids were successfully prepared via the dissolution–regeneration process of cellulose using a LiBr aqueous solution. Activated carbon suspended in a cellulose solution dissolved by heating with a LiBr aqueous solution was embedded into a gel directly formed by lowering the temperature of the cellulose solution. The cellulose-activated carbon gels exhibited large specific surface areas and sufficient mechanical properties. The adsorption capacity of methylene blue onto the cellulose-activated carbon gels proportionally increased with the increasing content of activated carbon. The cellulose-activated carbon gels maintained a high adsorption capacity even after repeated adsorption–desorption cycles, demonstrating their potential as reusable adsorbents.

## 1. Introduction

Industrial effluents from dyes, heavy metals, oils, and organic compounds cause serious environmental problems due to their toxicity, persistence, and bioaccumulation [1,2,3,4]. Various methods for removing pollutants from wastewater, including adsorption, precipitation, oxidation/reduction, coagulation/flocculation, membrane filtration, and biological degradation, have been used to treat waste effluents [5,6].

Adsorption is an effective separation technique due to its low cost, simplicity of design, ease of operation, efficiency, and reusability of adsorbents [7,8]. Among various adsorbents, activated carbon has attracted considerable attention for the removal of pollutants from wastewater because of its high specific surface area and porosity, low density, structural variety, and thermal and chemical stability [9,10,11]. The economics of the adsorption process using activated carbon depends on the easy reusability of activated carbon [12,13]. However, it is difficult to separate the activated carbon from the solution after adsorption due to the small particle size of activated carbon. Recovering powdered activated carbon inevitably requires high-speed centrifugation or filtering [12,14].

Cellulose is the most abundant renewable organic substance on earth. In addition, linear structures of cellulose molecules exhibit strong mechanical properties via the numerous inter- and intra-molecular hydrogen bonds of its anhydroglucose repeating units. These special characteristics of cellulose have led to extensive interest in the development of cellulose-based composites in combination with natural and synthetic polymers, metals, and metal oxides [15,16,17,18]. To prepare cellulose-based composites, cellulose solvents such as *N*-methylmorpholine-*N*-oxide, LiCl/*N*,*N*-dimethylacetamide, alkali/urea aqueous solution, and ionic liquids have been used [19,20,21,22]. For advanced applications, composites can be prepared in various forms of films, membranes, hydrogels, aerogels, fibers, and so on. In particular, a number of studies on the removal of dyes and heavy metals using cellulose-based composites have been performed [23,24,25].

One of the many aqueous cellulose solvents, a LiBr aqueous solution, has the unique characteristic of facilitating direct regeneration of the dissolved cellulose [26,27,28]. Cellulose, completely dissolved in a concentrated LiBr aqueous solution by heating, can be directly gelled from the solution by cooling without a non-solvent. In a previous study, cellulose–SiO_2_ gels with a high SiO_2_ content up to 90% were successfully developed via the mixing of SiO_2_ particles and a cellulose solution dissolved in a LiBr aqueous solution [29]. Also, a variety of forms of SiO_2_-embedded gels could be easily prepared using the direct gelation of a cellulose solution dissolved in a LiBr aqueous solution. Therefore, this dissolution–regeneration system is expected to be effective for trapping activated carbon particles into regenerated cellulose forms. The development of activated carbon-embedded cellulose gels with a high activated carbon content will facilitate the recovery of activated carbon particles and improve their reusability as adsorbents.

In this study, the dissolution–regeneration of cellulose in a LiBr aqueous solution system was applied to prepare cellulose gels with embedded activated carbon. The effects of activated carbon content on the morphologies, specific surface areas, bulk densities, porosities, and mechanical properties of the gels were investigated. The potential applicability of the cellulose-activated carbon gels as adsorption materials for hazardous dye removal were investigated using methylene blue as a model dye. In addition, the reusability of the cellulose-activated carbon gels was evaluated by repeated adsorption–desorption experiments.

## 2. Materials and Methods

### 2.1. Preparation of Cellulose-Activated Carbon Gels

First, 2 g of cellulose (viscosity-average degree of polymerization 890, filter paper pulp, Advantec MFS, Dublin, CA, USA) and a certain amount (0.50–4.67 g) of commercial activated carbon particles (AC, particle size < 100 mesh, Sigma-Aldrich, St Louis, MO, USA) were mixed in 98 g of a 60 wt% LiBr aqueous solution. By controlling the initial weight ratios of cellulose and AC, six grades of the cellulose-activated carbon gels (CAC20–CAC70) were prepared. The numbers following CAC represent the weight percentages of the AC relative to the total solids (AC + cellulose) in CACs. For example, the weight percentage of the AC in CAC70 was 70% (4.67 g of AC + 2 g of cellulose). The mixed solution was treated at 150 °C for approximately 10 min, and the dissolved solution was cast onto glass plates or poured into 24-well plates. The solution became a gel when cooled at room temperature. After the gels were thoroughly washed with deionized water, the hydrogels were treated via solvent exchange (ethanol → *tert*-butyl alcohol) and freeze-dried. For comparison, cellulose gel (CG) without AC was prepared using the same experimental procedure except with a dissolution time of approximately 20 min.

### 2.2. Determination of Activated Carbon Content

AC content was evaluated as the carbon contents by elemental analysis and the amounts of residual solid by thermogravimetric analysis. The elemental analysis was conducted at a combustion temperature of 900 °C with a thermal conductivity detection system (Flash EA 1112, CE Elantech, Farmingdale, NY, USA). AC contents were determined as the ratio of the analytical carbon contents of the CACs and the theoretical contents calculated based on those of CG and AC. Thermogravimetric analysis (TGA) was performed between room temperature and 500 °C at a heating rate of 10 °C/min in a nitrogen atmosphere (TGA N-1000, Scinco, Seoul, Republic of Korea). AC contents were determined as the ratio of the analytical amounts of residual solids of the CACs at 500 °C and the theoretical amounts calculated based on those of CG and AC.

### 2.3. Characterization

The bulk densities of the freeze-dried gels were determined by dividing weight by volume, and the porosity of the gels was examined by liquid displacement using hexane, as reported previously [30]. Nitrogen physisorption measurements at 77 K were performed for Brunauer–Emmett–Teller (BET) and Barrett–Joyner–Halenda (BJH) analyses (BELSORP-max, MicrotracBEL, Osaka, Japan). The BET analysis was performed at a relative vapor pressure of 0.05–0.3, and the BJH analysis was performed from the desorption branch of the isotherm. Field Emission Scanning Electron Microscope (FE-SEM) images were obtained using the freeze-dried gels fractured in liquid nitrogen to expose a cross-section that was coated with platinum using a sputter coater (MERLIN, Carl Zeiss, Oberkochen, Germany). X-ray diffractometry (XRD) measurements were performed at 40 kV and 40 mA with monochromatized and collimated Cu Kα radiation (λ = 0.15418 nm) via reflections from a parabolic multi-layer mirror (D8 Advance, Bruker, Billerica, MA, USA). Compressive tests were carried out using cylindrical freeze-dried gels with a 12 mm diameter and 14 mm height; the tests were stopped at a compression load of 135 N (Instron 5844, Instron Corp., Norwood, MA, USA). The compressive moduli were determined by drawing a parallel line starting at 2% strain. The pH of the point of zero charge (pH*_pzc_*) was investigated using a batch equilibrium experiment at a pH value of 5–10 using 0.1 M HCl or NaOH solutions, as reported previously [31].

### 2.4. Adsorption and Reusability Study

The adsorption properties of the CACs were examined using methylene blue (MB) in the batch adsorption experiments. The sample (approximately 20 mg) in the MB solution (10 mL) was shaken using a 2D rocker (30 rpm) at 25 °C in a thermos-hygrostat. The effect of pH was studied using various pH solutions (pH 5–10) adjusted with a 0.1 M HCl or NaOH solution for 24 h at an MB concentration of 500 mg/L. The effect of contact time was examined for different time intervals of up to 120 h at an MB concentration of 500 mg/L at pH 10. The effect of the initial dye concentration was investigated using various MB concentrations (50–4000 mg/L) at pH 10 for 72 h. The residual dye concentrations were analyzed using an ultraviolet spectrophotometer (Evolution 201, Thermo Scientific, Waltham, MA, USA) at 660 nm, and the amounts of dye adsorbed were calculated based on the following equation:

$$q_{t}=\frac{(C_{0}-C_{t})V}{m}$$

where *C*_0_ is the initial dye concentration (mg/L), *C_t_* is the dye concentration at time *t*, *V* is the solution volume (L), and *m* is the weight of the gel (g).

The reusability of the gel was examined by performing successive adsorption–desorption cycles. The adsorption experiment was carried out using CAC70 (approximately 20 mg) for an MB concentration of 100 mg/L (10 mL) at a pH of 10 for 24 h. After measuring the amount of adsorbed MB, the MB-loaded gel was treated with 50% acetone and washed several times with deionized water. The adsorption–desorption cycle was then repeated ten times.

## 3. Results and Discussion

### 3.1. Preparation of Cellulose-Activated Carbon Gels

**Figure 1:**
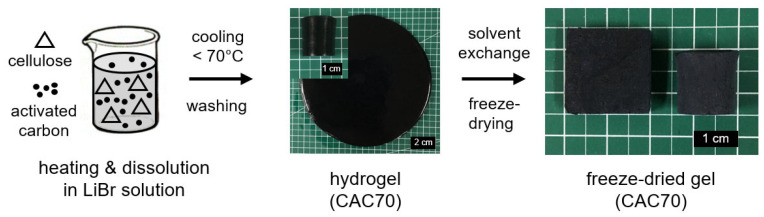
The preparation scheme of the cellulose-activated carbon gels using a LiBr aqueous solution.

Cellulose dissolved via heating in a LiBr aqueous solution is directly regenerated during the cooling process without the use of a non-solvent [26]. This unique characteristic of direct coagulation of dissolved cellulose can facilitate the preparation of various types of cellulose-based materials. In this study, this dissolution–regeneration system was applied to introduce activated carbon particles (AC) into a cellulose gel. A mixture of cellulose and AC in a 60 wt% LiBr aqueous solution was treated at 150 °C for approximately 10 min. The dissolved cellulose solution, which included AC, was rapidly regenerated by forming a gel in less than about 30 min while cooling below approximately 70 °C. Two types of gels, plate- and cylinder-shaped forms, could be easily prepared using the dissolution–regeneration system in a LiBr aqueous solution (Figure 1). Freeze-dried cellulose-activated carbon gels (CACs) were successfully obtained after the extraction of LiBr with deionized water and the replacement of water with alcohols (ethanol → *tert*-butyl alcohol).

The presence of AC shortened the dissolution time of cellulose compared with the dissolution time of approximately 20 min for cellulose without AC. When the treatment time was longer than approximately 10 min in the presence of AC, the viscosity of the cellulose solution was rapidly lowered in a short time, such that no gel could be formed. The same phenomenon was observed when the AC content was 80% or more. In this case, the viscosity of the cellulose solution dramatically decreased in a very short time even compared with the 70% AC content. Compared with the pH of 6.7 for a 60 wt% LiBr aqueous solution, the pH of the LiBr aqueous solution with AC was in the acidic range: 3.2 (CAC20), 2.5 (CAC30), 2.0 (CAC40), 1.8 (CAC50), 1.6 (CAC60), and 1.4 (CAC70). The high-temperature treatment in the acidic region must have affected the cleavage of glycosidic bonds of cellulose molecular chains, which resulted in a significant decrease in the degree of polymerization of cellulose to prevent the gelation of cellulose.

**Figure 2:**
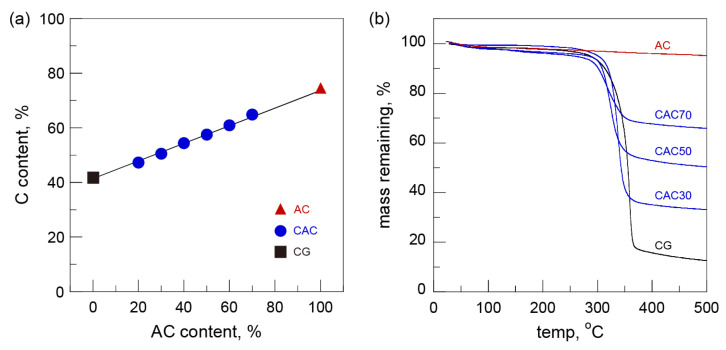
(**a**) Carbon (C) contents and (**b**) thermogravimetric curves of cellulose-activated carbon gels.

Elemental and thermogravimetric analyses were performed to investigate the AC content of the CACs. According to the results of the elemental analysis, the carbon (C) contents of CG and AC were 42.3% and 73.1%, respectively. The C content of the CACs showed a clear increase from 47.3% (CAC20) to 64.8% (CAC70) with the increase in AC content (Figure 2a). Also, the amounts of residual solids at 500 °C of the CACs (considering the moisture content) were in fairly good agreement according to the initial blend ratio of cellulose and AC (Figure 2b). These two values corresponded to above 98% of the amount of AC used. These results indicate that AC particles were tightly trapped inside the gels without any loss during the regeneration process.

### 3.2. Surface Area, Pore Size Distribution, Porosity, Bulk Density, and Morphology

**Figure 3:**
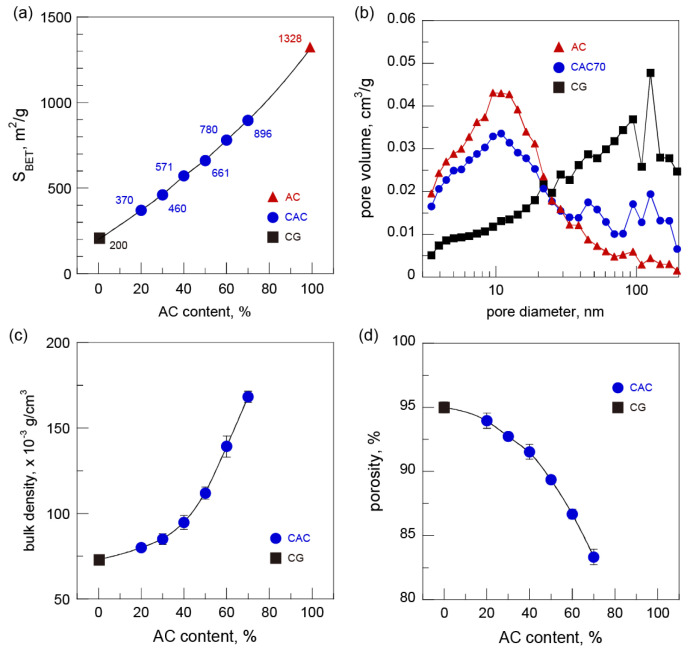
(**a**) Specific surface area, (**b**) BJH pore size distribution, (**c**) bulk density, and (**d**) porosity of cellulose-activated carbon gels.

Cellulose-based gels with a high specific surface area (S_BET_) could be easily prepared via the introduction of AC (S_BET_ 1328 m^2^/g). The S_BET_ values of the CACs gradually increased from 370 m^2^/g (CAC20) to 896 m^2^/g (CAC70) with the increasing AC content. The S_BET_ value of CAC70 was 4.5 times higher than that of CG (200 m^2^/g) (Figure 3a). However, the S_BET_ values of the CACs corresponded to approximately 86–91% of the theoretical S_BET_ values calculated based on the blend ratio of cellulose and AC. This result suggests that a small portion of the pores of AC were filled by the regenerated cellulose during the gelation process. However, the lower S_BET_ values of the CACs (as compared to the theoretical S_BET_ values) were not affected by the pore size distributions. The pore size distributions of CAC70 calculated from the BJH model were nearly a superposition of the pore size distributions of CG and AC, similar to AC with a pore size smaller than 20 nm and CG with a pore size above 20 nm (Figure 3b).

The changes in the bulk density and porosity due to the insertion of AC into the gel were investigated using the plate-shaped freeze-dried gels. With the increase in the AC content, the bulk density of the CACs distinctly increased up to 168 × 10^−3^ g/cm^3^ for CAC70, 2.3 times higher than that of CG (Figure 3c). The increased bulk density of the CACs also led to decreases in porosity. Compared with the approximately 95% porosity for CG, the porosity of the CACs decreased with the increasing AC content, resulting in about 83% porosity for CAC70 (Figure 3d). The changes in the void volumes of the CACs are likely due to the dense packing of AC particles into the composite gels rather than the introduction of a large amount of AC.

**Figure 4:**
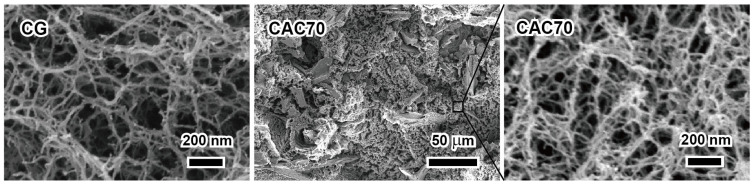
Cross-sectional FE-SEM images of cellulose-activated carbon gels.

To confirm the difference in the void volumes of CG and CACs, cross-sections of the freeze-dried gels were observed by FE-SEM (Figure 4). The regenerated cellulose matrix in CAC70 containing a large amount of AC had a three-dimensional porous structure consisting of a number of long nanofibrils. The sizes of pores in the regenerated cellulose matrix in CAC70 ranged about 20–150 nm wide, significantly smaller than those in CG. These results are also considered to be due to the dense packing of AC, and the decrease in the pore sizes in CAC agrees well the results of reduced porosity. Similar results were reported for cellulose–SiO_2_ gels prepared in a LiBr aqueous solution [29].

### 3.3. Structural, Thermal, and Mechanical Analysis

**Figure 5:**
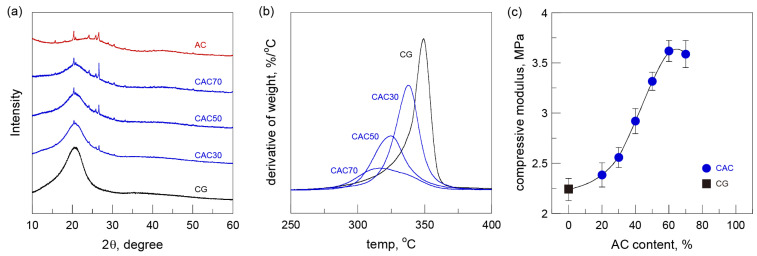
(**a**) X-ray diffraction spectra, (**b**) derivative thermogravimetric curves, and (**c**) compressive moduli of cellulose-activated carbon gels.

The structural changes during the preparation process of the gels were investigated using X-ray diffraction analysis (Figure 5a). The dissolution–regeneration of cellulose in a LiBr aqueous solution induced a structural change from highly crystalline cellulose I to poorly crystalline cellulose II, which featured a strong peak at 21° [26]. The X-ray diffraction pattern of AC showed several sharp peaks derived from metals composed of Ca, Fe, K, Mg, and Si, among others [32]. The X-ray diffraction patterns of the CACs exhibited combined patterns in proportion to the initial blend ratios of CG and AC, revealing no interference in the structural formation between the two components.

The thermal stabilities of the gels were examined in a nitrogen atmosphere. The thermal degradation of CG started at about 285 °C and rapid weight loss occurred at 310–360 °C. Although the thermal decomposition patterns of the CACs on the TG curves were similar to those of CG, the initial temperature of thermal decomposition and the range of weight loss of the CACs shifted to a lower temperature with the increase in the AC content (Figure 2b). The changes were more clearly seen in the derivative thermogravimetric curves (DTG) (Figure 5b). The DTG peaks of the CACs moved from 337 °C (CAC30) to 315 °C (CAC70) with the increasing AC content compared with the DTG peak of 350 °C for CG. These results are likely to be associated with the deterioration of thermal stability stemming from the reduced degree of polymerization of cellulose under severe dissolution processes such as high-temperature treatment in the acidic region.

The mechanical properties of the gels were investigated by compression testing using cylinder-shaped gels (12 mm in diameter and 14 mm in height). The compressive moduli determined at 2% strain in the linear elastic region are plotted versus AC content (Figure 5c). The compressive moduli of the CACs gradually increased with the increasing AC content despite the decreased content of the regenerated cellulose matrix. The highest value of the compressive modulus was observed in CAC60, which was about 1.6 times higher than that of CG. The increased compressive moduli of the CACs were highly dependent on the AC content, corresponding to their higher densities and lower porosities, as shown in Figure 3. However, the compressive modulus was slightly lower when the AC content reached 70% (CAC70). This result is attributed to the lower content of the regenerated cellulose matrix and the reduced degree of polymerization of cellulose.

### 3.4. Adsorption Study

AC is widely used as an adsorption material for various substances such as dyes, heavy metals, and organic compounds mainly due to its high specific surface area. In this study, the adsorption ability of the CACs was evaluated using a cationic dye, methylene blue (MB).

**Figure 6:**
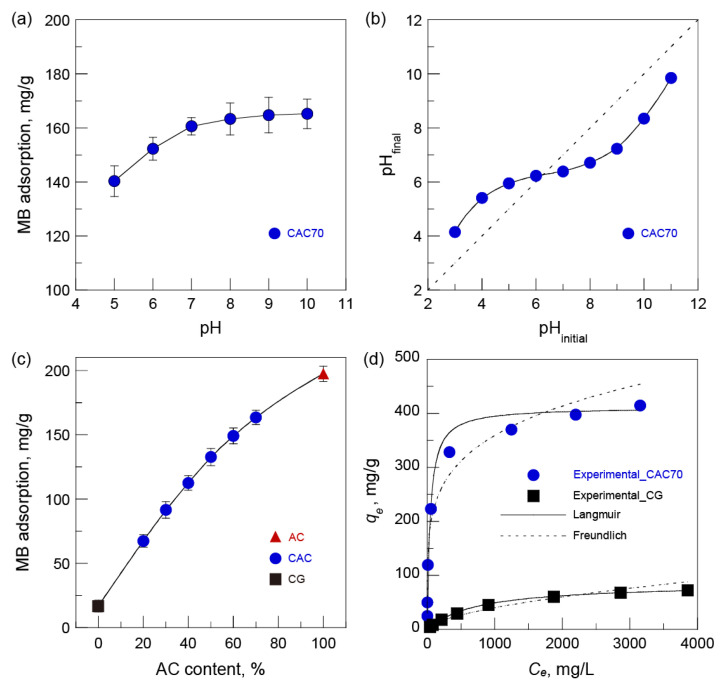
(**a**) The effect of pH on the adsorption of MB onto CAC70 (MB concentration: 500 mg/L; 24 h), (**b**) the pH of point of zero charge of CAC70, (**c**) the effect of AC content on the adsorption of MB (MB concentration: 500 mg/L; pH 10; 24 h), and (**d**) the MB adsorption isotherms of CG and CAC70 (MB concentration: 50–4000 mg/L; pH 10; 72 h).

First, the effect of solution pH on MB adsorption was investigated using CAC70 at pH values ranging from 5 to 10 (Figure 6a). The amount of MB adsorbed onto CAC70 gradually increased with the increase in pH up to 8 and was almost constant above pH 8. The solution pH has a decisive effect on both the surface charge of the adsorbent and the ionization of the dye adsorbate [12,33]. MB is dominantly dissociated in cationic forms at pH values ranging from 5 to 10 because the p*K*_a_ of MB is 3.8 [34]. The pH of the point of zero charge (pH*_pzc_*) of the CAC70 was 6.3 (Figure 6b), resulting in a positive surface charge when pH < pH*_pzc_* and a negative surface charge when pH > pH*_pzc_*. Therefore, the electrostatic interactions between the negative surface of the AC and the cationic forms of MB likely enhanced the adsorption capacity of MB in neutral and alkaline conditions. Nevertheless, the adsorption capacity of MB was still high under acidic conditions. This is because other factors such as van der Waals attraction, π-interactions of the aromatic dye molecules with the adsorbent surface, and chemical interactions may also play important roles in the adsorption of MB onto the CAC surface [35].

The effect of the AC content of CACs on MB adsorption was investigated at a pH of 10 where the fraction of both the negative surface of the AC and the cationic forms of MB was the largest (Figure 6c). The adsorption of MB onto the CACs proportionally increased with the increase in AC content. This result is attributed to an increase in the adsorption sites due to the increase in the surface area of the adsorbent [36]. However, the MB adsorption capacities of the CACs were approximately 21 mg/g higher than the theoretical adsorption capacities calculated based on the blend ratio of CG (16.7 mg/g) and AC (197.3 mg/g). Future detailed study on the variation in MB adsorption capacity is needed to assess the contribution of the cellulose matrix to dye adsorption.

To determine how adsorbates interact with adsorbents, an equilibrium study of MB adsorption was carried out at differential initial concentrations ranging from 50 to 4000 mg/L at a pH of 10 for 72 h (Figure 6d). The experimental data were fitted using the linear forms of Langmuir (Equation (1)) and Freundlich (Equation (2)) isotherms expressed as follows:

(1) $$\frac{C_{e}}{q_{e}}=\frac{1}{{K_{L}q}_{max}}+\frac{1}{q_{max}}C_{e}$$

(2) $$\ln q_{e}=\ln K_{F}+\frac{1}{n}\ln C_{e}$$

where *C_e_* (mg/L) is the equilibrium concentration of the MB solution, *q_e_* (mg/g) is the quantity of MB adsorbed at time *t*, *K_L_* (L/mg) is the Langmuir equilibrium constant, *q_max_* (mg/g) is the theoretical maximum adsorption capacity, *K_F_* (mg/g) is the Freundlich equilibrium constant, and *n* is the heterogeneity factor.

The Langmuir adsorption model is based on the monolayer adsorption of an adsorbate at the adsorbent surface containing a finite number of identical sites, resulting in the existence of a maximum absorption limit [35,37]. The Freundlich adsorption model is based on the multilayer adsorption of an adsorbate at the heterogeneous surface of the adsorbent, resulting in an increase in the degree of absorption according to adsorbate concentration [12,38]. From the regression values (R^2^) and two isotherms obtained from the equilibrium fitting data (Table 1 and Figure 6d), the Langmuir isotherm model demonstrates a better fit for both CG and CAC70 than the Freundlich isotherm model.

The introduction of AC remarkably increased the *q_max_* of CAC70 up to 411.3 mg/g, which is 4.8 times higher than 86.1 mg/g of CG. The remarkable difference in *q_max_* of CG and CAC70 indicates that AC could play a significant role in enhancing the adsorption of MB. The *q_max_* of CAC70 was lower than other research results using powdered AC: 613.8 mg/g with activated carbon prepared from Date Press Cake at pH 7 [35], 580 mg/g with bituminous coal-based activated carbon at pH 11 [37], and 454.2 mg/g with bamboo-based activated carbon at pH 7 [38].

**Figure 7:**
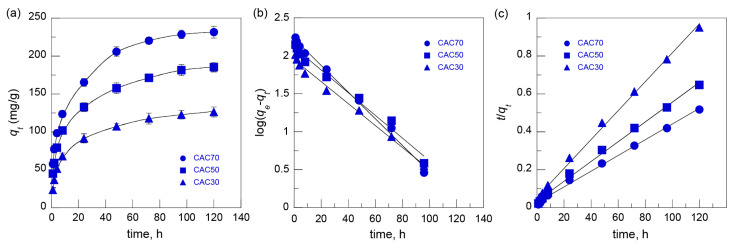
(**a**) The effect of contact time on the adsorption of MB onto CACs (MB concentration: 500 mg/L; pH 10) and adsorption kinetics by using (**b**) pseudo-first-order and (**c**) pseudo-second-order models.

The effect of contact time on MB adsorption was examined at an MB concentration of 500 mg/L at a pH of 10. The adsorption of MB onto the CACs increased rapidly within 8 h and then proceeded gradually for 120 h regardless of AC content (Figure 7a). The experimental kinetic data were fitted with pseudo-first-order (Equation (3)) and pseudo-second-order (Equation (4)) models (Figure 7b,c) expressed in the form of the following equations:

(3) $$\log\left(q_{e}-q_{t}\right)=\log q_{e}-\frac{k_{1}}{2.303}t$$

(4) $$\frac{t}{q_{t}}=\frac{1}{k_{2}q_{e}^{2}}+\frac{1}{q_{e}}t\;\mathrm{and}\;h=k_{2}q_{e}^{2}$$

where *q_e_* (mg/g) is the amount of MB adsorbed at equilibrium, *q_t_* (mg/g) is the amount of MB adsorbed at time *t*, *k*_1_ (min^−1^) is the pseudo-first-order constant, *k*_2_ (g mg^−1^ min^−1^) is the pseudo-second-order constant, and *h* (mg g^−1^ min^−1^) is the initial adsorption rate.

The correlation coefficients (R^2^) of the pseudo-second-order model were higher than those of the pseudo-second-order model, and the theoretical *q_e_* values of the pseudo-second-order model were close to the experimental values for all CACs (Table 2). These results indicate that the adsorption process for MB onto CACs followed that of the pseudo-second-order model, suggesting that the overall adsorption kinetics were governed by the chemisorption process dependent on both MB and CAC [39,40]. The *h* values reveal that the initial adsorption rate increased with the increase in the AC content of the CACs.

### 3.5. Reusability

**Figure 8:**
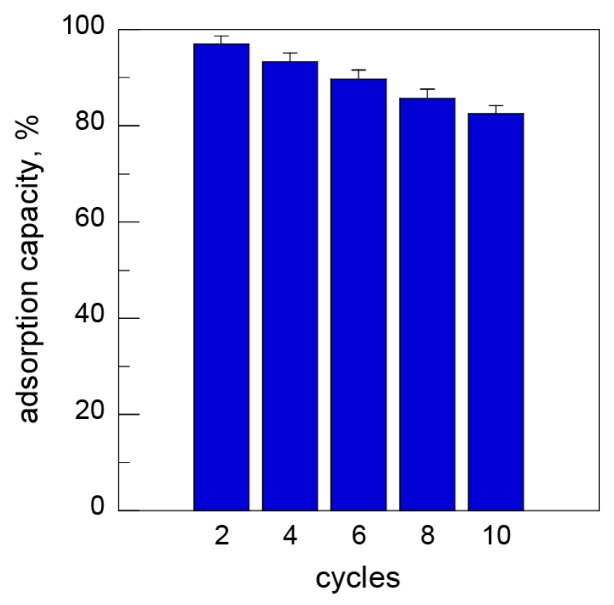
Adsorption capacity of CAC70 for MB adsorption over ten cycles (MB concentration: 100 mg/L; pH 10).

It is difficult to separate powdered AC from a solution after adsorption because of the small particle sizes of AC. The cellulose-activated carbon gels prepared in this study were expected to be suitable for recycling because the AC particles were tightly embedded into the gels. Therefore, to investigate the reusability of the cellulose-activated carbon gel as an adsorbent, the adsorption–desorption cycles were repeated ten times using CAC70 with an MB concentration of 100 mg/L at a pH of 10 (Figure 8). Although the adsorption capacity of CAC70 gradually decreased during each cycle, CAC70 still maintained 82.5% of its original adsorption capacity after ten cycles. These results indicate that the cellulose-activated carbon gel has excellent regeneration properties and significant potential for repeated use as an adsorbent.

## 4. Conclusions

Cellulose-activated carbon gels with a high activated carbon content were successfully prepared via the dissolution–regeneration process of cellulose using a LiBr aqueous solution. Direct gel formation by lowering the temperature of the cellulose solution dissolved by heating facilitated the introduction of activated carbon particles into the gels. Unfortunately, the maximum activated carbon content in cellulose-activated carbon gels was 70%, which represents the limit of activated carbon for the gelation of cellulose solutions under high-temperature treatment in the acidic region. Although the cellulose-activated carbon gels included a high activated carbon content, they had sufficient mechanical strength. The specific surface area of the cellulose-activated carbon gels was improved to 896 m^2^/g, making them highly effective for the adsorption of methylene blue with an adsorption capacity of 411.3 mg/g. Repeated adsorption–desorption cycles showed that the adsorption capacity of the cellulose-activated carbon gels was 82.5% after ten cycles. Therefore, cellulose-activated carbon gels are expected to be suitable for application as adsorbents in the field of wastewater purification.

## Figures and Tables

**Table 1:** Adsorption isotherm constants of Langmuir and Freundlich models.

Sample	Experimental *q_max_* (mg/g)	Langmuir			Freundlich		
		*q_max_* (mg/g)	*K_L_* (×10^3^)	R^2^	*n*	*K_F_*	R^2^
CG	72.8	86.1	1.365	0.9974	1.73	0.757	0.9783
CAC70	414.6	411.3	23.64	0.9980	4.91	88.04	0.9824

**Table 2:** Kinetic parameters for MB adsorption onto CAC (MB concentration: 500 mg/L; pH 10).

Sample	*q*_*e* exp_ (mg/g)	Pseudo-First-Order			Pseudo-Second-Order			
		*q_e_* (mg/g)	*k*_1_ × 10^−2^ (min^−1^)	R^2^	*q_e_* (mg/g)	*k*_2_ × 10^−4^ (g mg^−1^ min^−1^)	R^2^	*h* (mg g^−1^ min^−1^)
CAC30	126.3	89.1	3.33	0.9913	131.3	10.61	0.9974	18.30
CAC50	185.4	129.7	3.45	0.9844	191.8	7.85	0.9963	28.87
CAC70	231.5	168.2	4.05	0.9931	241.6	6.43	0.9972	37.52

## Data Availability

The data are contained within the article.

## Abbreviations

The following abbreviations are used in this manuscript:

CAC	cellulose-activated carbon gel
CG	cellulose gel
AC	activated carbon
TGA	thermogravimetric analysis
DTG	derivative thermogravimetric curve
BET	Brunauer–Emmett–Teller
S_BET_	specific surface area
BJH	Barrett–Joyner–Halenda
FE-SEM	Field Emission Scanning Electron Microscope
XRD	X-ray diffractometry
pH*_pzc_*	pH of point of zero charge
p*Ka*	Acid dissociation constant
MB	Methylene blue
